# Computational account for the naturalness perception of others’ jumping motion based on a vertical projectile motion model

**DOI:** 10.1098/rspb.2024.1490

**Published:** 2024-09-18

**Authors:** Takumi Yokosaka, Yusuke Ujitoko, Takahiro Kawabe

**Affiliations:** ^1^ NTT Communication Science Laboratories, Kanagawa, Japan

**Keywords:** naturalness perception, human action, computational model, jumping motion, motion prediction, gravity

## Abstract

The visual naturalness of a rendered character’s motion is an important factor in computer graphics work, and the rendering of jumping motions is no exception to this. However, the computational mechanism that underlies the observer’s judgement of the naturalness of a jumping motion has not yet been fully elucidated. We hypothesized that observers would perceive a jumping motion as more natural when the jump trajectory was consistent with the trajectory of a vertical projectile motion based on Earth’s gravity. We asked human participants to evaluate the naturalness of point-light jumping motions whose height and duration were modulated. The results showed that the observers’ naturalness rating varied with the modulation ratios of the jump height and duration. Interestingly, the ratings were high even when the height and duration differed from the actual jump. To explain this tendency, we constructed computational models that predicted the theoretical trajectory of a jump based on the projectile motion formula and calculated the errors between the theoretical and observed trajectories. The pattern of the errors correlated closely with the participants’ ratings. Our results suggest that observers judge the naturalness of observed jumping motion based on the error between observed and predicted jump trajectories.

## 1. Introduction

When observing character actions rendered using computer graphics, such as in films and games, these actions can sometimes seem unnatural. The mechanism of how observers perceive an agent’s action as being natural is an important topic, both from the perspective of understanding human computational mechanisms for perceiving others’ motion and from the perspective of designing natural agent action. Regarding the perception of an agent’s action, a number of studies using biological motion have assessed the ability of an observer to estimate an agent’s properties, such as gender [1,2], identity [3–5], emotional state [6] and effort exerted to pull an object [7]. Regarding research into the naturalness of agent action, an earlier study reported that a Bayesian model trained on actual walking motion using movement primitives was able to generate a natural walking action [8]. Another line of study [9] examined the effect of modifying throwing action appearances on the perceptual plausibility of the action and reported that shorting the distance of an underarm throw was judged to be less plausible than an overarm one. On the other hand, the modification of throwing action speeds did not influence the plausibility, compared with the modification of ball speeds. Another study reported that participants had difficulty in detecting the modulated angular momentum of observed acrobatics performed by a character [10].

**Figure 1.**
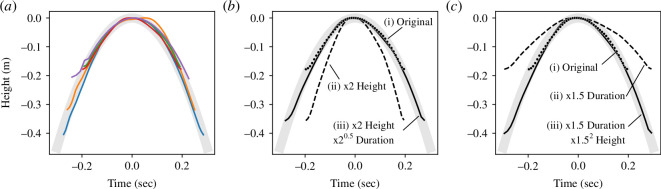
Height–time trajectories of jumping actions. Thick grey lines indicate the theoretical trajectory *h* calculated from the formula $h=v_{0}t-\frac{1}{2}gt^{2}$ , where *t*, *g* and *v*_0_ represent time, Earth’s gravity and initial velocity, respectively. In this figure, the height was calculated such that the maximum height of a jump for each jumper was set as 0 on the *y*-axis, and the time was calculated such that the point in time when each jumper reached the maximum height of each jump was set as 0 on the *x*-axis. (*a*) Coloured lines denote trajectories of five jumping actions extracted from a motion capture database. (*b*) Example of height modulation in the motion capture data. (*i*) The original jump trajectory, (ii) the height-modulated trajectory due to the height being multiplied by *H* and (iii) the height- and duration-modulated trajectory due to the duration being multiplied by $\sqrt{H}$ . (*c*) Example of duration modulation in the motion capture data. (*i*) The original jump trajectory, (ii) the duration-modulated trajectory due to the duration being multiplied by *T* and (iii) the duration- and height-modulated trajectory due to the height being multiplied by $T^{2}$ .

Here, we focus on the naturalness perception of a common human motion, the act of jumping. A previous study [11] investigated how observers recognized human jumping actions by decomposing jumping actions into translation and deformation components and found that the translation component made a greater contribution to an observer’s evaluation of the jump than the deformation component. The translation component of a jumping action on Earth can be expressed as a vertical projectile motion based on Earth’s gravity $h=v_{0}t-\frac{1}{2}gt^{2}$ . Figure 1*a* shows the height–time trajectories of five jumpers extracted from a motion capture database (see §2c for more details). These five jumping trajectories can be seen to overlap the trajectory calculated from the formula for a vertical projectile motion h (shown by the thick grey line in figure 1*a*).

A number of earlier studies have discussed the ability of humans to predict the motion of objects using prior knowledge of gravitationally accelerated motion based on sensory experience [12,13]. For example, as if predicting a gravitationally accelerated object’s motion, experimental participants have been demonstrated to track an object with their eye movement [14–17], to intercept the fall of an object with their hands [18–25], and to estimate the duration of the motion [26–28] and the velocity of the object [29]. Earlier studies have also reported neurophysiological evidence for internal representations of gravity [30–36]. Note that it is not clear whether people’s prior knowledge of gravity comes from their actual perception of gravity itself, as some studies suggest that people do not need to perceive gravity to determine the direction of balance [37,38]. Overall, participants are able to accurately interact with falling objects, suggesting that they are using their *a priori* knowledge of gravity on Earth.

Although these studies have mainly focused on the motion of a rigid object like a ball, a similar computational mechanism for gravitationally accelerated motion may be involved in the perception of the naturalness of human body movement. In this line of inquiry, some studies have demonstrated the involvement of gravity in the perception of biological motion. For example, the perception of biological motion has been shown to be impaired for inverted point-light walker stimuli [39,40], a phenomenon thought to be due to a gravity bias [41]. Changes in the actual gravitational environment around the observer reduce the inversion effect [42]. Observers can also estimate the weight of a box lifted by a point-light actor by utilizing gravitational cues provided by the postural change of the actor and the acceleration of the box [43]. Changes in the gravity level of ballistic motions are easier to detect with a human character than with a ball character [44]. Thus, while researchers have accumulated findings on the detection and recognition of biological motion being dependent on knowledge of gravity, little is known about how natural observers perceive the jump action as a gravitational event.

We hypothesize that the (in)congruence of trajectories between an observed jump motion and a theoretical projectile motion in the vertical direction plays an important role in the perception of the naturalness of a human jumping action. We assume that observers predict a jump trajectory using information about the jumper’s initial motion and prior knowledge about gravitationally accelerated object motion. It is expected that observers’ perception of the naturalness of jump actions will be more impaired as the jumper’s jump trajectory differs more widely from the theoretical jump trajectory.

According to our hypothesis, even if we modulated the trajectory of a recorded jumping action so that it differed from the actual trajectory but remained congruent with the theoretical trajectory, observers should perceive a high degree of naturalness in the modulated jumping action. By way of example, we illustrate the modulated jumping trajectory in figure 1*b,c*. When we modulate the height of a recorded jumping action (figure 1*b*(i)) by multiplying it by H , the trajectory of the height-modulated jumping action deviates from that calculated by the formula $h=v_{0}t-\frac{1}{2}gt^{2}$ (figure 1*b*(ii)). This height-modulated jumping action was expected to be perceived as unnatural by observers. Calculations from the formula for vertical projectile motion show that if the height is multiplied by H , the height–time trajectory will match the formula $h=v_{0}t-\frac{1}{2}gt^{2}$ when the duration of the jumping action is also multiplied by $\sqrt{H}$ (figure 1*b*(iii)). If, instead of the height, the duration of the recorded jump action is multiplied by T , the height–time trajectory will match the formula $h=v_{0}t-\frac{1}{2}gt^{2}$ when the height of the jumping action is also multiplied by $T^{2}$ (figure 1*c*). These modulated jumping actions featuring no deviations from the theoretical trajectory were expected to be perceived as the most natural by the observers.

This hypothesis may not be correct since, as shown by studies in the field of intuitive physics (or naïve physics) [45], humans do not always estimate the falling motion of an object based on the laws of physics. For example, when asked where to drop an object to hit a target on the ground while the object is in a moving carrier, people fail to use the correct frame of reference [46,47]. When judging the speed of fall, despite the clearly established physical principle of the mass–velocity relationship, people often labour under a misbelief that a heavier object falls faster than a lighter one [48,49]. Some people mistakenly believed that a thrown ball would continue to accelerate and judged distorted ball trajectories as natural [50]. Recent studies showed that this misbelief was not only related to mental imagery but also extended to the judgement of actual visual events [51,52], and even the naturalness and animacy impressions for the visual events. For example, observers feel a more natural impression when the larger objects descend faster [53,54]. In addition to gravitational events, it has also been reported that pendulum motions that differ from those observed in the real world are perceived as natural [54–56]. Studies investigating the animacy impression in the context of naïve physics have reported that dots bouncing under less than Earth’s gravity induce the physical bounce impression, while dots bouncing under Earth’s gravity induce the animated motion impression [57]. In summary, a body of research in naïve physics has accumulated evidence that observers do not necessarily judge the visual events of objects falling under Earth’s gravity to be physically correct or natural events following Newtonian principles. If an intuitive sense of physical principles is dominant in judging the naturalness of a human’s jumping motion, a jump trajectory may not seem natural to observers, even if it follows the formula $h=v_{0}t-\frac{1}{2}gt^{2}$ .

In this study, we test the hypothesis that the greater the congruence between the observed and theoretical trajectories of a jumping action, the higher the naturalness rating observers will assign to it. In §2, we report the procedures and results of an experiment in which participants were asked to rate the naturalness of jumps modulated by a height modulation ratio H ( H = 0.50−2.01) and a duration modulation ratio T ( T = 0.50−2.01). We obtained naturalness ratings for each combination of H and T . Note that our approach tests participants’ broader understanding of gravitational acceleration by generating jumps with a variety of accelerations, which differs from the approach used in several previous studies that tested participants’ *a priori* knowledge of terrestrial gravitational acceleration using interaction tasks [18–25]. In §3, we test a naturalness model using the distance between the actual and theoretical modulation ratios. In §4, we test alternative models using the difference between the modulated and theoretical trajectories. In §5, we test models inspired by previous studies investigating the human perception of gravitationally accelerated objects. Finally, in §6, we discuss the computational mechanisms underlying the perception of naturalness in a human jumping action and the limitations of the proposed models.

## 2. Experiment

We conducted an online experiment to investigate how observer ratings of the naturalness of modulated point-light jumper stimuli change according to the applied modulation ratios (height modulation ratio *H* and duration modulation ratio T ).

### (a) Participants

To balance the gender and age attributes of the participants, we recruited five participants for each of the following six attributes: (female and male) × (20s, 30s and 40s). Each of the five participants was assigned to one of five different jumper stimuli (see §2c). A Japanese crowdsourcing research company recruited the participants online and they were paid for their participation. Although we expected to obtain response data from a total of 30 participants, two additional participants participated in an experiment before the corresponding experimental URL was closed, so that in the end we obtained response data from 32 participants. The mean age ± s.d. of the 32 participants (16 females and 16 males) was 34.37 ± 8.57.

The participants were unaware of the specific purpose of the experiment. Ethical approval for this study was obtained from the ethics committee at Nippon Telegraph and Telephone Corporation (approval number: R02-009 by NTT Communication Science Laboratories Ethics Committee). The experiments were conducted according to principles that have their origin in the Helsinki Declaration excluding the pre-registration. Written informed consent was obtained from all observers in this study.

### (b) Apparatus

The experiments in this study were carried out online with participants using their own personal computers (PCs) and mice. Because our experimental script could only be run on a PC, devices with neither a physical keyboard nor a mouse, such as smartphones or tablets, could not be used in this experiment.

### (c) Stimuli

**Figure 2.**
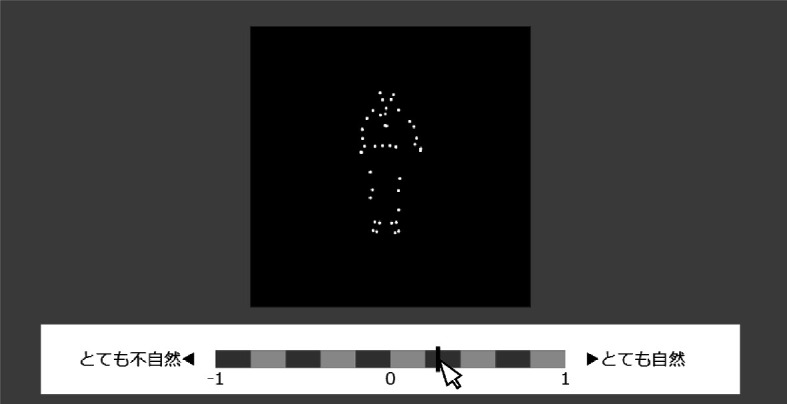
Experimental set-up. The phrase to the right of the visual analogue scale (VAS) reads ‘very natural’ in Japanese. The phrase to the left of the VAS reads ‘very unnatural’ in Japanese.

The experimental stimuli consisted of a human point-light figure jumper and a visual analogue scale (VAS), which are both shown in figure 2. We used full-body motion capture data from the Carnegie-Mellon Graphics Lab Motion Capture Database (http://mocap.cs.cmu.edu/). We adopted five jumper’s jumping action motion data variants from the database (the ‘subject trial’ numbering of the selected motion data variants was ‘02 04’, ‘13 39’, ‘16 01’ ‘49 02’ and ‘88 11’). We obtained the motion data in c3d format and converted them to csv files using the pyc3dserver library. Each set of motion data consisted of 41 point-light markers on a human body (see figure 2). Although each marker has a three-dimensional value of *x*, *y* and *z*, we used only the *x* and *y* values to render the jump action from a frontal viewing position. We provide details on how the jump height was modulated in electronic supplementary material, chapter A.

We generated and presented these stimuli using p5js. All point-light jumpers were drawn so that the total body height of each jumper in the first frame was equal to 50 mm on the screen of each participant’s monitor. We controlled the spatial dimension of these stimuli in millimetres by calculating the size of one pixel on each participant’s PC screen (see §2d for more details).

Because we planned to calculate the Spearman’s correlation coefficient between the participants’ naturalness ratings and the predictions obtained from the model, the total number of combinations of height modulation ratios H and duration modulation ratios T applied to the jump motion stimuli needed to be carefully determined to obtain reliable outcomes for the calculation. Using a statistical calculator, GPower, we computed the number of conditions that were required for the reliable outcomes of the calculation based on the following three criteria: a medium effect size (*r* = 0.3), a power of 80% and an alpha of 5%. The number of conditions that met these criteria was 82. Using this calculation as a reference, we adopted 81 conditions (i.e. combinations of nine height modulation ratios and nine duration modulation ratios). The nine modulation ratios were: exp(−0.7) = 0.50, exp(−0.525) = 0.59, exp(−0.35) = 0.70, exp(- 0.175) = 0.84, exp(0) = 1.00, exp(0.175)=1.19, exp(0.35)=1.42, exp(0.525)=1.69 and exp(0.7)=2.01. We modulated the jump height and duration by multiplying the translation component by the height modulation ratio H and the frame-times by the duration modulation ratio T , respectively. Examples of jump stimuli can be viewed in the electronic supplementary material, movie. Since we focused on differences in naturalness between modulation ratios rather than between jumpers, we presented each participant with only one of the five jumpers. In each trial, participants repeatedly observed the same point-light jumper completing a jump action until they had selected a naturalness rating on the analogue scale. A blank period of 0.5 s was shown on the screen between repeated presentations of the same jump.

Participants performed the rating task by clicking on a VAS (figure 2). To reduce the response bias, we created the VAS with a banded design [58] with three labels. Participants clicked on any position on the VAS and the location of the click was recorded as a data point on a 100-point scale with a minimum value of 0 and a maximum value of 1.

### (d) Procedure

We wanted to equalize the stimulus size across the participants’ monitors, which were expected to have different spatial resolutions in pixels. Therefore, before the formal test, we asked the participants to adjust the size of a rectangle on the screen with the left and right keys so that the rectangle was the same size as a credit card or a card of equal size held against the screen, following the method used by Li *et al*. [59]. Because the method assumes that the participants’ monitors have square pixels, participants were asked to adjust only the length of one side of the rectangle. We controlled the stimulus size in millimetres based on the measured screen pixel size. After that, to control the distance between the screen and the participants’ eyes, we instructed the participants to observe the screen from a distance of 600 mm.

At the beginning of the experiment, participants were given their instructions: ‘An animation of a jumping person will be drawn with white dots’ and ‘Please answer to what extent you think the speed of the jump action looks natural using the VAS’. We have referred here to the naturalness of ‘the speed of the jump action’ rather than the naturalness of ‘the jump action’ *per se* in order to compare the results with those of earlier studies that have examined motion perception with a focus on the speed of the movement. We also expected that this instruction would help prevent participants from judging the naturalness of the appearance of the point-light stimulus. After reading the instructions, they moved on to a practice session consisting of five conditions: an original condition (*H* = 1.00 and *T* = 1.00) and four extremely modulated conditions (*H* = 0.50 and *T* = 0.50, *H* = 0.50 and *T* = 2, 01, *H* = 2.01 and *T* = 0.50 and *H* = 2.01 and *T* = 2.01). The five conditions were presented in a randomized order. After completing all five trials of the practice session, the participants moved on to a session consisting of the main trials. In the main trials, 81 conditions (i.e. nine height modulation ratios × nine duration modulation ratios) were presented in a randomized order.

### (e) Data analysis

We used Python 3.0 for the model computations and the statistical processing of the naturalness ratings. The naturalness rating values were averaged across all 32 participants for each combination of height and duration modulation ratios.

### (f) Results of naturalness rating

**Figure 3.**
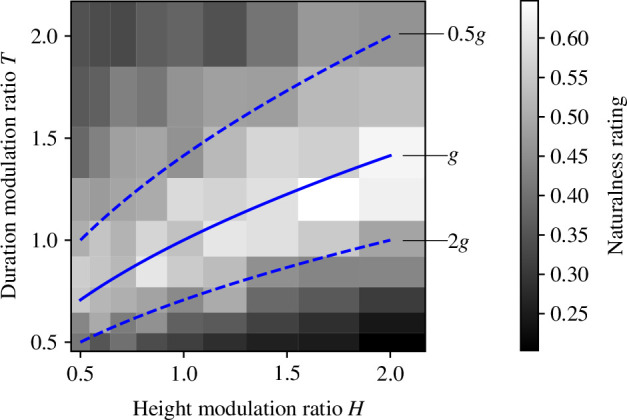
Results of naturalness ratings. The blue lines indicate theoretical curves ( $T=\sqrt{H}$ ) for three conditions of gravitational acceleration.

Figure 3 shows the results of the averaged naturalness ratings for each combination of height and duration modulation ratios. While rating values can range from 0 to 1, the average rating values across participants in figure 3 are in a relatively narrow range from 0.2 to 0.65. This result is due to the averaging of participants’ rating values with different variances; however, this statistical process does not seem to strongly affect the subsequent calculation of correlation coefficients with models (see electronic supplementary material, chapter B ). The results in figure 3 appear to show that the naturalness ratings tended to be high along the theoretical curve (the blue solid line; $T=\sqrt{H}$ ) calculated by the equation of vertical projectile motion. This result highlights two interesting points. The first is that observers appear to give higher ratings of naturalness for conditions where the relationship between the height modulation ratio and the duration modulation ratio follows the theoretical values, even when the modulation ratios are not always equal to one. The second interesting point is that the naturalness ratings tended to be lower when a jumper performed a higher jump over a shorter period of time (e.g. *H* = 2.01 and *T* = 0.50) than when a jumper performed a lower jump over a longer period of time (e.g. *H* = 0.50 and *T* = 2.01).

In addition to the tendency for naturalness ratings to be higher for conditions close to the theoretical curve (blue solid line in figure 3), we also found a tendency for naturalness ratings to be higher for conditions with larger modulation ratios than for conditions with smaller modulation ratios. This trend suggests that even under the same gravitational acceleration, participants rate higher and longer jumps as more natural than lower and shorter jumps. A possible reason for the tendency could be attributed to the difference in stimulus durations between the stimuli. Participants might not be able to obtain enough information from lower and shorter jumps to judge their naturalness due to the shorter duration of these jump motions (see also electronic supplementary material, video). The effect of stimulus durations on the amount of information obtained from the stimuli may also explain the preference for small acceleration/deceleration values when judging the impression of physical bouncing [57]. In §§3–5, we attempt to build a model of naturalness ratings that can account for these features.

## 3. Model 1: deviation from theoretical modulation ratios

### (a) Model analysis

As we explained in §1, the equation of vertical projectile motion ( $h=v_{0}t-\frac{1}{2}gt^{2}$ ) shows that the modulated jump trajectories follow a theoretical vertical projectile motion that reflects the effects of gravity when the duration of the jump is multiplied by $\sqrt{H}$ and the height is multiplied by H (or by $T^{2}$ when the duration is multiplied by T ). Here, we proposed a model of naturalness ratings by introducing the degree of deviation *d* of the modulation ratios used in the experiment from the theoretical ones. The degree of deviation $d_{H,T}$ for a height modulation ratio *H* and a duration modulation ratio *T* was defined by the following equation:


(3.1) $$\begin{array}{c} d_{H,T}=\left|log\left(T\right)-log\left(\sqrt{H}\right)\right|, \end{array}$$

where we took the logarithm of the modulation ratio as we assumed that the participants’ sensory response is non-linear with respect to the physical modulation ratio of height and duration.

### (b) Results and discussion

**Figure 4.**
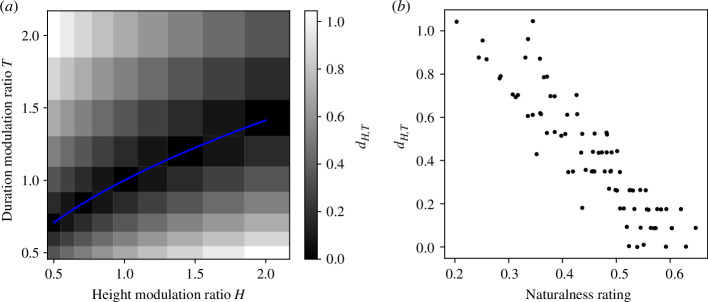
The model of deviation from the theoretical modulation ratios. (*a*) $d_{H,T}$ calculated from equation( 3.1). The blue line denotes a theoretical curve ( $T=\sqrt{H}$ or $H=T^{2}$ ). (*b*) A scatter plot of naturalness ratings and $d_{H,T}$ .

Figure 4b shows a scatter plot of the naturalness ratings and $d_{H,T}$ calculated from equation (3.1). The correlation coefficient *r* between them was −0.90 (*p* < 0.001; electronic supplementary material, figure C1). Histograms of the correlation coefficients for individual participants are shown in electronic supplementary material, figure D1 and show a relatively consistent tendency across participants. The correlation coefficients did not differ statistically significantly between any two of the jumper stimuli (electronic supplementary material, figure E1). This large negative correlation suggests that the degree of deviation from the theoretical modulation ratios can explain the observers’ ratings of naturalness. However, the model does not seem to explain some samples well. In figure 3, the naturalness ratings tended to be lower when a jumper performed a higher jump over a shorter period of time (e.g. *H* = 2.01 and *T* = 0.50) than when a jumper performed a lower jump over a longer period of time (e.g. *H* = 0.50 and *T* = 2.01). The model using the deviation from the theoretical modulation ratios could not replicate this difference, resulting in the forked tongue-like shape of the scatter plot in the 0.2–0.4 naturalness rating range in figure 4b. The observation that fast jumps are more unnatural than slow jumps suggests a mechanism weighting the faster jump action with a higher degree of deviation. However, this model does not contribute much to the discussion of the computational mechanisms by which human observers judge naturalness because what observers perceive when observing a point-light jumper is the optical motion, not the modulation ratio itself. In §4, we propose an alternative model that focuses on deviation in height, velocity and acceleration trajectories.

## 4. Model 2: matching predictive and modulated trajectories

The first model (based on the degree of deviation from the theoretical modulation ratios) could not explain well the tendency for naturalness ratings to be lower for faster jump actions than for slower jump actions. Here, we proposed alternative models that take into account the height–, velocity– and acceleration–time trajectories of the jump actions. These models assume that when a jump action is observed, the observer predicts a theoretical trajectory with the same initial velocity as that of the observed jump action and estimates the extent to which the observed trajectory matches the predicted theoretical trajectory. We hypothesized that the trajectories used for matching would be velocity trajectories rather than height and acceleration trajectories since in the experiment the naturalness ratings were lower for faster jump actions than for slower jump actions.

### (a) Model analysis

**Figure 5.**
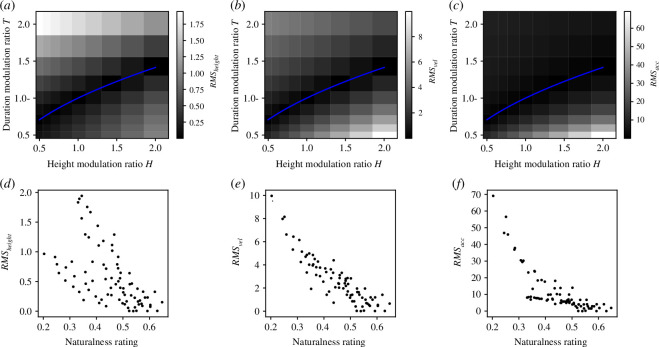
Model of predictive matching. (*a–c*) Each panel represents (*a*) RMS_height_, (*b*) RMS_vel_ and (*c*) RMS_acc_ for each condition with height modulation ratio *H* and duration modulation ratio *T*. Blue lines denote theoretical curves. (*d–f*) Scatter plots of naturalness ratings and (*d*) RMS_height_, (*e*) RMS_vel_ and (*f*) RMS_acc_.

The model is based on the following assumptions: (i) the observer extracts the initial velocity $v_{0}$ of the modulated jump; (ii) the observer predicts the height $h_{predict}$ , velocity $v_{predict}$ or acceleration $a_{predict}$ of the jumper using the initial velocity $v_{0}$ extracted in the previous step and the theoretically correct equation for gravitational motion and (iii) the observer evaluates the naturalness based on the disparity ${RMS}_{height}$ , ${RMS}_{vel}$ or ${RMS}_{acc}$ between the predicted theoretical trajectory $h_{predict}$ , $v_{predict}$ or $a_{predict}$ and the observed modulated jump h , v or a as follows:


(4.1) $$\begin{array}{c} RMS_{height}=\sqrt{\frac{\sum {\left(h-h_{predict}\right)}^{2}}{N}}, \end{array}$$

(4.2) $$\begin{array}{c} RMS_{vel}=\sqrt{\frac{\sum {\left(v-v_{predict}\right)}^{2}}{N}}, \end{array}$$

(4.3) $$\begin{array}{c} RMS_{acc}=\sqrt{\frac{\sum {\left(a-a_{predict}\right)}^{2}}{N}}, \end{array}$$

where *N* is the length of the sampled trajectory. For a more detailed derivation, see electronic supplementary material, chapter F. The RMS_height_, RMS_vel_ and RMS_acc_ in each condition with height modulation ratio *H* and duration modulation ratio *T* are shown in figure 5*a–c*.

### (b) Results and discussion

Figure 5*d-f* show scatter plots of naturalness ratings and RMS_height_, RMS_vel_ and RMS_acc_. The correlation coefficients between them were −0.58(*p *< 0.001), −0.89(*p *< 0.001),−0.79(*p *< 0.001), respectively (electronic supplementary material, figure C1). We tested whether the differences in the correlation coefficients between the ${RMS}_{height}$ , ${RMS}_{vel}$ and ${RMS}_{acc}$ were different from 0 by calculating 10 000 bootstrap samples of the differences with participant replacement and found significant differences for all combinations (Bonferroni-corrected *p *< 0.001). Histograms of the correlation coefficients for individual participants are shown in electronic supplementary material, figure D2 and show a relatively consistent tendency across participants for RMS_vel_ and RMS_acc_, but not for RMS_height_. The correlation coefficients did not differ statistically significantly between any two of the jumper stimuli for each model (electronic supplementary material, figure E1). The model that calculates the deviation in velocity trajectories between modulated and predicted jumps (RMS_vel_) shows the highest correlation with the experimental participant’s naturalness ratings. A notable feature of this model RMS_vel_ is that, as seen in figure 5*b,e*, it replicates well the tendency of naturalness ratings to be lower for faster jump actions than for slower jump actions. The model based on height trajectories RMS_height_ does not seem to replicate this trend (figure 5*d*) and the model based on acceleration trajectories RMS_acc_ seems to make the slower jumps relatively more natural as the model weights the naturalness too low for the faster jumps.

On the other hand, the correlation coefficient *r* = −0.89 of the proposed model RMS_vel_ was not improved compared to the correlation coefficient *r* = −0.90 of the model based on the deviation $d_{H,T}$ of the modulation ratio in §3. This lack of improvement might be due to the greater discrepancy between the predictions from RMS_vel_ and the naturalness ratings in the higher range of naturalness ratings compared to the lower range. That is, the discrepancy likely results from a tendency for naturalness ratings to be higher for conditions with larger modulation ratios than for conditions with smaller modulation ratios, as discussed in the previous chapter. It is inferred that the RMS_vel_ model does not account for the effect of stimulus durations on the amount of information obtained from the stimuli.

One might wonder whether the Earth’s gravitational acceleration *g* is the optimal value when calculating the predicted trajectory. Indeed, previous studies have suggested that gravitational motions with acceleration values less than *g* are perceived as more natural than those with acceleration values equal to or greater than *g* [52,57]. However, the results of the correlation calculations that varied the values of the gravitational acceleration from 0.5*g* to 2*g* showed that the negative correlation between RMS_vel_ and naturalness ratings was greatest when the gravitational acceleration was close to 1*g* (see electronic supplementary material, chapter G for details). The results suggest that participants might use the Earth’s gravitational acceleration *g* fairly accurately when judging the naturalness of the jumps made by the point-light jumper.

## 5. Model 3: difference in duration or maximum height of jumping action

Finally, we tested models inspired by previous studies investigating the human perception of gravitationally accelerated objects. Some studies have suggested that human observers estimate the duration of objects’ motion using their knowledge of the Earth’s gravitational acceleration [17,27]. In experiments, participants observed an object moving along a parabolic trajectory with varying acceleration that would then disappear in the middle of the motion. The participants were required to indicate when they thought the object would have returned to its initial height by clicking a mouse. These studies proposed a model in which the timings provided by the participants’ answers could be simulated from the velocity of the object just before it disappeared, the gravity of the Earth and the remaining distance the object had to cover when it disappeared. Applying this idea to our experimental situation, we can propose a model in which an observer predicts the specific timing of the jump (e.g. when the jump will reach the highest point or when the jump will be completed) and compares it with the timing of a modulated jump. For example, given the initial velocity of a modulated jump *v*′_0_, the gravity of the Earth *g* and the velocity–time formula $v=v_{0}-gt$ , the time at which the jump reaches the highest point can be predicted as v0`g . We can also consider an alternative model that predicts the maximum height of the modulated jumps. In this model, given the initial velocity of a modulated jump *v*′_0_, the gravity of the Earth *g* and the velocity–height formula $v^{2}-v_{0}^{2}=-2gh$ , the maximum height can be predicted as v0`22g .

### (a) Model analysis

The analysis procedure was the same as in §4, up to the point where the initial velocity *v*′_0_ of the modulated jump was calculated. When a jumper performs a jumping action with the initial velocity *v*′_0_ under the gravity of the Earth, the theoretical time in which the jumper will reach the maximum height is v0`g and the actual time in which the jumper reaches the maximum height is tnT2 . Thus, the difference in the theoretical and actual times $\tau_{diff}$ can be calculated as follows:


(5.1) $$\begin{array}{c} \tau_{diff}=|\frac{v_{0}^{\prime }}{g}-\frac{t_{n}T}{2}|. \end{array}$$

Instead of the time in which the jumper reaches the maximum height, we can calculate the $\tau_{diff}$ for the time in which the jumper completes the jumping action as follows:


(5.2) $$\begin{array}{c} \tau_{diff}=|\frac{2v_{0}^{\prime }}{g}-t_{n}T|. \end{array}$$

The correlation coefficients with the naturalness rating are the same for both models. We use the model in equation (5.1) in the subsequent analysis.

The theoretical maximum height is v0`22g and the actual maximum height of the modulated jump is max(*h*′(*t*′′_*i*_)). Thus, the difference in the theoretical and actual heights $h_{diff}$ can be calculated as follows:


(5.3) $$h_{diff}=|\frac{v_{0}^{\prime 2}}{2g}-\max\left(h^{\prime }\left(t_{i}^{\prime \prime }\right)\right)|.$$

**Figure 6.**
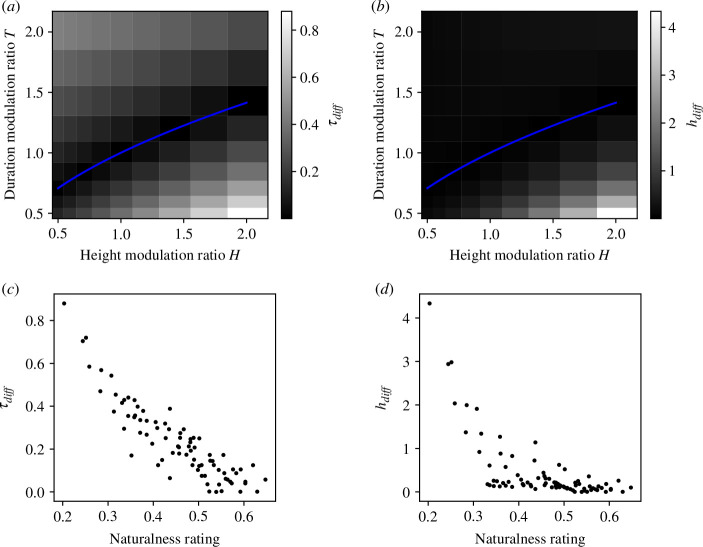
(*a*) $\tau_{diff}$ for each condition with height modulation ratio *H* and duration modulation ratio *T*. (*b*) $h_{diff}$ for each condition with height modulation ratio *H* and duration modulation ratio *T*. (*c*) Scatter plots of naturalness ratings and $\tau_{diff}$ . (*d*) Scatter plots of naturalness ratings and $h_{diff}$ .

The $\tau_{diff}$ and $h_{diff}$ are shown in figure 6*a,b*, respectively.

### (b) Results and discussion

Figure 6*c,d* shows scatter plots of naturalness ratings and $\tau_{diff}$ and $h_{diff}$ . The correlation coefficients between them were −0.89(*p *< 0.001) and −0.68(*p *< 0.001), respectively (electronic supplementary material, figure C1). We tested whether the difference in the correlation coefficients between the $\tau_{diff}$ and $h_{diff}$ was different from 0 by calculating 10 000 bootstrap samples of the differences with participant replacement and found a significant difference (*p *< 0.001). Histograms of the correlation coefficients for individual participants are shown in electronic supplementary material, figure D3 and show a relatively consistent tendency across participants. The correlation coefficients did not differ statistically significantly between any two of the jumper stimuli for each model (electronic supplementary material, figure E1). The results show that the model that calculates prediction errors for specific timings during the jump better explains observers’ naturalness ratings than the model that calculates prediction errors for the maximum height. Interestingly, the correlation coefficients of the difference between the theoretical and actual time $\tau_{diff}$ was the same as that for RMS_vel_. Indeed, from the velocity–time formula $v=v_{0}-gt$ , the relationships between $\tau_{diff}$ and RMS_vel_ can be calculated as follows:


(4.7) $$\begin{array}{c} \tau_{diff}=\frac{\sqrt{3}}{2g}RMS_{vel}, \end{array}$$

where RMS_vel_ was calculated as follows:


(4.8) $$\begin{array}{c} RMS_{vel}=\sqrt{\frac{\int_{0}^{t_{n}T}{\left(v^{\prime }\left(t\right)-v_{predict}\left(t\right)\right)}^{2}dt}{t_{n}T}}. \end{array}$$

Since *g* is a constant, the difference in velocity trajectories (RMS_vel_) and the prediction error in jump duration $\tau_{diff}$ are theoretically linearly proportional. This calculation suggests that, in the context of our stimulus manipulation, it is impossible to distinguish whether human observers compute errors in jump trajectories sequentially or only the difference in jump duration. To disentangle these possibilities, a modulated jump stimulus with the same duration as the theoretical trajectory predicted by the initial velocity, but with a dynamic velocity change that differs from the theoretical trajectory, might be useful.

We also tested whether the differences in the correlation coefficients between model 1, ${RMS}_{vel}$ in model 2,and $\tau_{diff}$ in model 3 were different from 0 by calculating 10 000 bootstrap samples of the differences with participant replacement and found no significant difference (Bonferroni-corrected *p *> 0.05). These results suggest that these models may to the same extent explain participants’ naturalness ratings.

## 6. General discussion

### (a) Mechanism of naturalness perception for human jumping action

In the psychophysical experiment, participants observed a jumping action performed by a human point-light figure, whose height and duration were independently modulated, and rated the naturalness of the jumps. We proposed naturalness models that computed the difference between observed and theoretical jumps. The results show that the observers’ naturalness ratings are highly correlated with the models.

Our results give insight into the computational mechanism responsible for the naturalness judgement of an observed human jump. All the models presented above describe the difference between an observed jump and a theoretical jump derived from formulas of a vertical projectile motion based on the Earth’s gravity and could generally explain the observers’ naturalness ratings well. These results suggest that humans compare observed and theoretical jumps. The mechanism underlying estimations of the gravitationally accelerated motion of a rigid body might also be involved in the prediction of theoretical jump trajectories. There is some evidence suggesting the ability of humans to predict the motion of objects using prior knowledge of gravitationally accelerated motion based on sensory experience [12,13]. Although the actual human jumping action is a complex motion in which body parts move to some extent independently [11], observers may regard the human in motion as a rigid body.

Here, we discuss the computational mechanisms of the naturalness perception by discussing models 2 and 3 (rather than model 1, which focuses on the modulation ratio). Model 2 assumes that an observer (i) predicts the jump trajectory according to gravitational acceleration from the initial velocity of the observed jump and (ii) continuously computes the difference between the predicted and observed trajectories of jumps. A model using the difference between velocity trajectories correlated most strongly with observer naturalness ratings, rather than models that used height or acceleration trajectories. The results suggest that observers might predict the velocity trajectory of a jump based on the initial velocity of the observed jump and perform a comparison of the predicted and actual velocity trajectories. The prediction of velocity trajectory for error computation has been reported, for example, in studies of self-motion estimation during cursor movement [60,61]. Therefore, a mechanism that predicts the plausible velocity trajectory based on the initial velocity of the observed jump and continuously monitors the error between the prediction and the velocity trajectory of the observed jump might be involved in naturalness perception.

Note that the velocity monitoring hypothesis cannot be distinguished from the duration estimation hypothesis for the present experimental stimuli. The models proposed in §5 compute the maximum height of the jump, or the time to maximum height, from the initial velocity. The difference between the predicted and observed time to maximum height explained the naturalness ratings better than the difference between the predicted and observed maximum height, and was also analytically linearly proportional to the difference between the velocity trajectories. This suggests that, at least for our stimulus manipulation, it is impossible to distinguish whether human observers continuously compute errors in velocity trajectories or only the difference in jump duration. Models have been proposed to predict the timing of the fall of a gravitationally accelerated object without continuous monitoring of the velocity trajectory: the model uses knowledge of size and gravity combined with the object’s visual angle and elevation angle [62,63]. To understand how human observers compute naturalness (which strategy they use or whether they use a different strategy), it will be necessary to design alternative experimental set-ups in which these hypotheses diverge. One possible experimental design is to use jump stimuli in which the duration matches what is predicted from the initial velocity and projectile motion, but the velocity trajectory deviates from the prediction. If $\tau_{diff}$ can sufficiently explain the naturalness ratings for the stimuli, human observers do not seem to need to compute the difference in velocity trajectories.

### (b) Possible explanation with direct perception

We assume that the perception of the naturalness of other people’s jumps involves a sequence of computational processes in the brain, wherein the jumper’s motion pattern in the retinal image is detected and the difference between the jumper’s motion pattern and an internal model of the vertical projectile motion is calculated. From a perspective different from the one adopted in our study, one might consider that the naturalness of jumps is not the result of the brain’s computation, but rather a direct perception in which an observer directly perceives the naturalness of jumps in the external world. Direct perception accounts can exclude any form of computation and account for all aspects of perception. As examples of direct perception, studies have suggested the existence of kinematics that specify various dynamics of other people, such as a weight being lifted [43], pulling force [7], person properties [64], communicative or deceptive expressions [64], maximum and preferred sitting heights [65], maximum reach-with-jump height [66], and so on (see [67] for a review). In the context of jump execution, previous studies have also discussed the visual information that jumpers can directly use to determine the vertical lift of their steps during a striding motion [68,69]. It might also be possible to interpret our results as a direct perception of the naturalness of projectile motion by humans, based on differences in the structure of stimuli with different spatio-temporal parameters. For example, the spatiotemporal structure of our stimuli might contain information representing the differences between the theoretical and observed trajectories. Further investigation is required to determine whether the perception of the naturalness of jumps is based on computation in the brain or a direct perception of information contained in the spatiotemporal stimulus structure.

### (c) Possible limitation and applicability

One possible limitation of the current models is that they do not take into account the relationship between jumper size and jump height. For example, a 10 m jump by a 1.8 m tall jumper would seem unnatural as the jumping action is not humanly possible. Even if the trajectory of this impossible jump matched the theoretical trajectory, the observer would have a greater impression of unnaturalness. Thus, our model might not be able to explain observers’ ratings of naturalness when the height modulation ratio is much larger than the stimulus used in our experiment (i.e. *H* > 2.01). This possible limitation leads us to an interesting hypothesis that the observer might acquire prior knowledge of the jump height range based on the size of the jumper and use this knowledge in the naturalness judgement. Experiments examining naturalness ratings for different jumper sizes in the monitor and height modulation ratio *H* would be useful to test this hypothesis.

In this study, we used human point-light figure jumpers as stimuli since we were interested in the translation component of the jump. However, if the appearance of the jumper is highly realistic, several factors other than the vertical projectile motion could affect the naturalness ratings. For example, when a character wearing clothes jumps, air resistance will deform the clothes. Since air resistance is proportional to the speed of the object, a higher theoretical (i.e. faster) jump will cause more deformation of the clothes than a lower theoretical (i.e. slower) jump. Therefore, when a high jump is modulated to become a low jump, observers might perceive it as unnatural since the large deformation of the clothes would not match that predicted by the low jump (or the inconsistency between jump height and deformation of the clothes might affect the perceived material of the clothes rather than any aspect of the jump motion itself). Other factors, such as the facial expression of the jumper and the apparent weight of the clothes the jumper is wearing, might also influence the perceived naturalness of the jump.

In our experiments, we excluded preparatory/landing actions from the stimuli as we wanted to focus only on the trajectory of the vertical projectile motion. Therefore, it is unclear whether these excluded actions can influence an observer’s perception of the naturalness of the jump. In composing animation works, the expressive technique of ‘anticipation’ is often used. This sort of technique to emphasize the preparatory action is often used to give the observer a sense of the jumper’s effort. Even if the jump trajectory is consistent with the theoretical trajectory, observers may find it unnatural if the preparatory action and/or landing action are not consistent with the jumping action (for example, a very small jump after a long preparatory action might give observers an impression of unnatural motion). One direction for future research would be to investigate the relationships between the movement during the jump itself and the preparatory/landing action.

### (d) Possible contribution to motion design

An interesting finding of this study is that even modulated jump action from motion capture data can be perceived as natural by observers as long as the trajectory of the jump matches the theoretical trajectory. Further investigation of the applicability of the present findings may provide design guidelines for the rendering of character motion in computer graphics. Creators often use motion capture to record human body movements and move computer-generated characters based on these movements. Creators often need to edit the motion capture data to be consistent with the surrounding environment, for example by adjusting the height of the jump so that the character cannot climb over surrounding structures. According to our findings, one way to adjust the jump height while maintaining the naturalness of the character’s jumping action is to also multiply the jump duration by the square root of the height modulation ratio. Another possible application of our findings is the intentional creation of unnatural jumps. Creators could design a jump trajectory that deviates from the vertical projectile motion based on Earth’s gravity to emphasize the jumper’s superhuman motor skills. Thus, providing a certain plausible model for the perception of the naturalness of a character’s movements can contribute to motion design.

## Data Availability

The datasets supporting this article have been uploaded as part of the supplementary material [70].
